# Activated Carbon/Transition Metal (Ni, In, Cu) Hexacyanoferrate Nanocomposites for Cesium Adsorption

**DOI:** 10.3390/ma12081253

**Published:** 2019-04-16

**Authors:** Julien Kiener, Lionel Limousy, Mejdi Jeguirim, Jean-Marc Le Meins, Samar Hajjar-Garreau, Gaetan Bigoin, Camélia Matei Ghimbeu

**Affiliations:** 1 Institut de Science des Matériaux de Mulhouse (IS2M) UMR 7361, Université de Haute-Alsace, CNRS, F-68100 Mulhouse, France; julien.kiener@yahoo.com (J.K.); Lionel.limousy@uha.fr (L.L.); Mejdi.jeguirim@uha.fr (M.J.); Jean-Marc.Le-meins@uha.fr (J.-M.L.M.); samar.hajjar@uha.fr (S.H.-G.); 2 Université de Strasbourg, F-67081 Strasbourg, France; 3 ONET Technologies, 36 Bd de l’Océan-CS 20280, 13258 Marseille, France; gbigoin@onet.fr

**Keywords:** potassium metal hexacyanoferrate, activated carbon, Cs adsorption, impregnation

## Abstract

Transition metal hexacyanoferrate/microporous activated carbon composites were obtained using a simple successive impregnation approach. The effect of metal type (nickel, indium, or copper), and the carbon oxidation on the composite characteristics (porosity, metal structure, and particle size), as well as on the removal efficiency of cesium from aqueous solution was investigated. Successful formation of the desired metal hexacyanoferrate phase was achieved and the size of the metallic nanoparticles and their dispersion in the carbon network was found to depend on the metal type, with the indium and nickel-based materials exhibiting the smallest particle size distribution (< 10 nm). Adsorption tests performed under batch conditions demonstrate that the copper hexacyanoferrate/activated carbon composite present the highest cesium removal capacity from aqueous solution (74.7 mg·g^−1^) among the three studied metal-based nanocomposites. The carbon oxidation treatment leads to the increase in the number of functional groups to the detriment of the porosity but allows for an improvement in the Cs adsorption capacity. This indicates that the Cs adsorption process is governed by the carbon surface chemistry and not its porosity. Moreover, combining oxidized carbon support with copper hexacyanoferrate induces the highest cesium adsorption capacity (101.5 mg·g^−1^). This could be related to synergistic effects through two absorption mechanisms, i.e., a cation exchange mechanism of Cs with the metallic hexacyanoferrate phase and Cs adsorption via carbon oxygen surface groups, as demonstrated using X-ray photoelectron spectroscopy (XPS) analyses.

## 1. Introduction

In case of a nuclear power plant accident, such as Fukushima-Daiichi and Chernobyl disasters, various radionuclides might be released into the environment. In fact, seawater used for the reactor cores cooling at Fukushima-Daiichi was contaminated with various radionuclides. Among them, uranium (^235^U) produces a high amount of ^135^Cs and ^137^Cs fission products, which have long half-lives (2 × 10^6^ years and 30.17 years, respectively), are highly radiotoxic and represent a serious threat for the environment. Moreover, due to the rather similar structure as potassium, it replaces potassium and gets easily incorporated in soil and water leading to the destruction of the eco-system. If ingested, Cs may induce severe diseases such as cancer [1]. Therefore, there is a great need to remove Cs from radioactive waste waters in order to avoid all these negative impacts on human and environment. Different techniques such as ionic exchange, reverse osmosis, adsorption removal, and sand filtration have been tested to remove Cs from drinking water [2]. Among these techniques, adsorption appears as a very effective and economical process. Thus, it appears necessary to synthesize new and efficient materials for cesium removal from water in case of contamination. In this context, different adsorbents have been tested such as zeolites, activated carbons, and transition metal hexacyanoferrate [3].

Activated carbons, having high surface area and developed porosity, are recognized as efficient for many pollutants captured [4]. Furthermore, activated carbons have a low cost and high mechanical strength and good resistance toward heat, chemicals, and radiation. These materials were among the first studied as cesium adsorbents [5]. A mixed adsorbent of chabazite, zeolite, and activated carbon has also been employed for the simultaneous removal of cesium and iodine from low-level liquid wastes [6]. Many works showed that the effective removal of cesium can be achieved with activated carbons obtained from raw materials such as sawdust [7,8], date pits [9], coconut shells [10], almond shells [11], or different commercially available activated carbons [4]. The main factors affecting the sorption behavior of Cs of these materials are the solution pH and concentration, the presence of some coexisting ions, and the adsorption temperature [8]. The materials characteristics may also impact the adsorption capacity.

Transition metal hexacyanoferrates (MHCFe) are another interesting class of materials that are efficient for Cs sorption due to their cubic structure having a size compatible with the diffusion of Cs ions. The performance of unsupported transition metal ferrocyanides have been evaluated for Cs^+^ removal from contaminated water. Copper hexacyanoferrate (CuHCFe) was the first studied for cesium sorption properties [12,13], but nickel hexacyanoferrate [14,15,16] and cobalt hexacyanoferrate [17] were also employed. While it is difficult to compare the performances of these materials due to the different conditions employed during cesium adsorption measurements, the maximum sorption properties have been attained by copper and nickel hexacyanoferrate materials [18]. It has been pointed out that these materials exhibit their best performances at a nanometer scale. However, manipulating nanoparticles induces complex problems regarding their recovery after cesium capture, which might lead to the dispersion of hazardous materials in water [18]. To overcome the problems due to the nanometer scale of transition metal ferrocyanides, a solution consists of using a support to immobilize them. Prussian blue (PB, iron hexacyanoferrate) nanoparticles have been tested while associated to cellulose nanofibers for their immobilization [19]. Composite materials combining transition metal hexacyanoferrate nanoparticles and a porous framework, such as zeolites [20,21] and mesoporous silica [22,23], appear very interesting for such applications. Vo et al. [24] obtained nanocrystals (2 nm) of potassium-cobalt hexacyanoferrate by using mesoporous silica MCM-41 as a support. Chitin has been also used as a support for different metal hexacyanoferrates [18], the maximum sorption capacities being attained using copper and nickel hexacyanoferrate. Montmorillonite clay has been also studied as a support for copper hexacyanoferrate and the maximum sorption capacity reached 206 mg·g^−1^.

Among these composite materials, the combination of activated carbon with transition metal hexacyanoferrate is of a particular interest due to the sorption capacity of both components. Shiozaki et al. directly impregnated Prussian blue on an activated carbon framework [25]. Hong et al. [26] synthesized a three-dimensionally ordered porous carbon to support Prussian blue nanoparticles via ultrasonic irradiation of FeCl_3_ and K_3_[Fe(CN)_6_] solution. The materials were used for a biomedical purpose, i.e., for the elimination of Cs from intestinal and oesophagus tract contaminated human body. The maximum ^133^Cs adsorption capacity was 40.07 mmol·g^−1^. Kawatake et al. [27] used two successive impregnations of K_4_[Fe(CN)_6_] and FeCl_3_ to obtain a Prussian blue/activated carbon composite. The Cs adsorption capacity reached 10.4 µmol·g^−1^ at the equilibrium Cs concentration of 49 µmol·dm^−3^. A potassium copper hexacyanoferrate/activated carbon material was obtained using a precipitation reaction of copper sulfate (CuSO_4_) with potassium hexacyanoferrate (K_4_[Fe(CN)_6_]) [28]. The same process was also used to synthesize a nickel hexacyanoferrate/activated carbon [29] composite. Lalhmunsiama et al. [30] proposed the use of biowaste, such as rice hulls and nut waste, to prepare activated carbon as support for nickel hexacyanoferrate, which exhibit a maximum Cs sorption capacity of 31.25 mg·g^−1^. Alternatively, Jeerage et al. [31] used cathodic deposition of nickel hexacyanoferrate on a carbon electrode for electrochemical sorption/desorption of Cs.

In this present investigation, a simple two-steps impregnation is proposed to design new nanocomposites materials combining highly porous commercial activated carbon (L27W) and different transition metals (nickel, indium, and copper) hexacyanoferrate. The adsorption performances of these synthesized nanocomposite materials are evaluated for the removal of cesium ion from aqueous solution. In addition, the influence of activated carbon surface chemistry on the nanocomposites adsorption efficiency is also studied. The adsorption mechanism is discussed in terms of the materials porosity, surface chemistry, and metallic phase. The combination of functionalized carbon support with metal hexacyanoferrate is demonstrated to be an efficient way to improve the Cs sorption capacity through different mechanisms.

## 2. Materials and Methods

### 2.1. Materials Synthesis

All metal salt precursors were purchased from Strem Chemical Inc. (Newburyport, MA, USA) and used without any modification: nickel nitrate hexahydrate Ni(NO_3_)_2_·6H_2_O (purity: 99.5%), indium nitrate hydrate In(NO_3_)_3_·xH_2_O (purity: 99.999%), copper nitrate trihydrate Cu(NO_3_)_2_·3H_2_O (purity: 99.5%), potassium ferricyanide K_3_[Fe(CN)_6_] (HCFe(III), purity: 98.5%), and potassium ferrocyanide K_4_[Fe(CN)_6_] (HCFe(II), purity: 99.0%).

Activated carbon L27W (purchased from Norit N.V (Amersfoort, Netherlands) was chosen as a support for metal hexacyanoferrate phases. Due to the presence of phosphorous residues from the chemical activation process, L27W was preliminary washed. During this step, 10.0 g of L27W were washed with distilled water heated at 70 °C, then dried in an oven (UF30 Memmert GmbH, Büchenbach, Germany) at 80 °C overnight (the obtained carbon was labeled C) Another carbon support with modified surface chemistry was prepared using a nitric acid (HNO_3_) treatment as follow: 5.0 g of L27W carbon, previously washed, was introduced in 200 mL of nitric acid (5 M) and the mixture was heated at 80 °C under reflux for 6 hours. Finally, the activated carbon was rinsed abundantly with water until reaching neutral pH. This oxidized carbon was labeled as C-ox.

Four activated carbon/metal hexacyanoferrate nanocomposites were synthesized: C/NiHCFe by combing Ni and HCFe(III), C/InHCFe by combining In and HCFe(II), C/CuHCFe by combining Cu and HCFe(II), and C-ox/CuHCFe obtained by applying a nitric acid treatment on L27W carbon.

The two-step impregnation process was carried out as follows:

Five grams of L27W, previously washed, was introduced in a 50 mL aqueous solution of metal (Ni, In, or Cu) nitrate at a molar concentration of 100 mM and mechanically agitated at room temperature for 24 hours. The nanocomposite was recovered via filtration, washed with water (≈200 mL) at room temperature and dried overnight in an oven at 80 °C.

The as-obtained nanocomposite was introduced in a 50 mL aqueous solution of potassium ferricyanide (100 mM) over 24 hours for the C/NiHCFe nanocomposite while a 50 mL aqueous solution of potassium ferrocyannide (100 mM) was used for C/CuHCFe and C/InHCFe nanocomposites. The rest of the process (rinsing-washing-rinsing-drying) was identical.

A last sample named C-ox/CuHCFe was synthesized following the same steps as for the C/CuHCFe but an activated carbon L27W preliminarily treated with nitric acid was used as the support. 

### 2.2. Materials Characterization

Data were collected with a powder diffractometer D8 ADVANCE A25 from Bruker (Billerica, MA, USA) in Bragg–Brentano reflexion geometry θ–θ. This diffractometer was equipped with the LynxEye XE-T high resolution energy dispersive 1-D detector (Cu Kα_1_,_2_). Data were collected from 10° up to 90° 2θ, with step size: 0.01° 2θ, and time per step: 0.5 s. Nitrogen adsorption/desorption was measured at 77 K using an ASAP 2420 from Micromeritics (Norcross, GA, USA). Pore and micropore volum were calculated using density functional theory (DFT) techniques with a slit pore model. The specific Brunauer–Emmett–Teller (BET) surface area (S_BET_) was calculated in the relative P/P_0_ domain: 0.01–0.05. The total pore volume (V_total pores_) and the microporous volume (V_micropores_) were calculated with DFT method. Mesoporore volume (V_mesopores_) was calculated by subtracting V_micropores_ from V_total pores_. The micropore size distribution was determined using a 2D-NLDFT (non-local density functional theory) heterogeneous surface model for carbon materials with slit pores implemented in SAIEUS (Micromeritics) [32]. Transmission electron microscopy (TEM) and scanning transmission electron microscopy (STEM) pictures were obtained on a ARM200 apparatus from JEOL (Peabody, MA, USA). EDX mapping was obtained with a JED 2300 (JEOL, Peabody, MA, USA) detector connected to the same microscope. (X-ray photoelectron spectroscopy) XPS data were measured with a VG Scienta SES-2002 spectrometer equipped with a concentric hemispherical analyser (Scienta Omicron, Uppsala, Sweden). The incident radiation used was generated by a monochromatic Al Kα X-ray source (1486.6eV) operating at 420 W (14 kV, 30 mA). A wide scan spectrum (survey) signal was recorded with a pass energy of 500 eV and for high resolution spectra pass energy was set to 100 eV.

Thermogravimetric analyses (TGA) were performed with a Mettler-Toledo TGA/DSC 3+ (Mettler-Toledo, Columbus, OH, USA) heating at 900 °C with a 10 °C/min heating ramp under air flow in order to determine the metal oxide residue in the nanocomposites. The results are given on a dry basis (without water contribution).

### 2.3. Cesium Adsorption Tests

Cesium adsorption capacities were measured using an atomic absorption spectroscope (AA 240 FS, Varian, Palo Alto, CA, USA). The wavelength used in atomic absorption for the cesium detection was 852.1 nm. Cesium nitrate (CsNO_3_) was chosen as the precursor in the contact solution and its concentration was fixed at 2 mmol/L (265 mg·L^−1^). The measurements were performed at room temperature under agitation for a contact time of 24 hours. A calibration of cesium concentration has been done to allow for further quantification. The nanocomposite concentration used in the contact solution was fixed at 1 g·L^−1^. Thereby, 50 mg of carbon or carbon/metal hexacyanoferrate nanocomposite and 50 mL of cesium stock solution were used. The adsorption capacities were calculated with the following equation:

q_e_ = (C_0_ − C_e_) × V/m

where C_0_ and C_e_ are the initial and equilibrium concentrations of Cs^+^ (mg·L^−1^) in the aqueous phase, respectively. V is the volume of the solution (L), m is the weight of adsorbent (g), and q_e_ is the adsorption amount of adsorbate per unit weight of adsorbent (mg·g^−1^).

## 3. Results and Discussion

### 3.1. Influence of the Transition Metal Type

**Figure 1:**
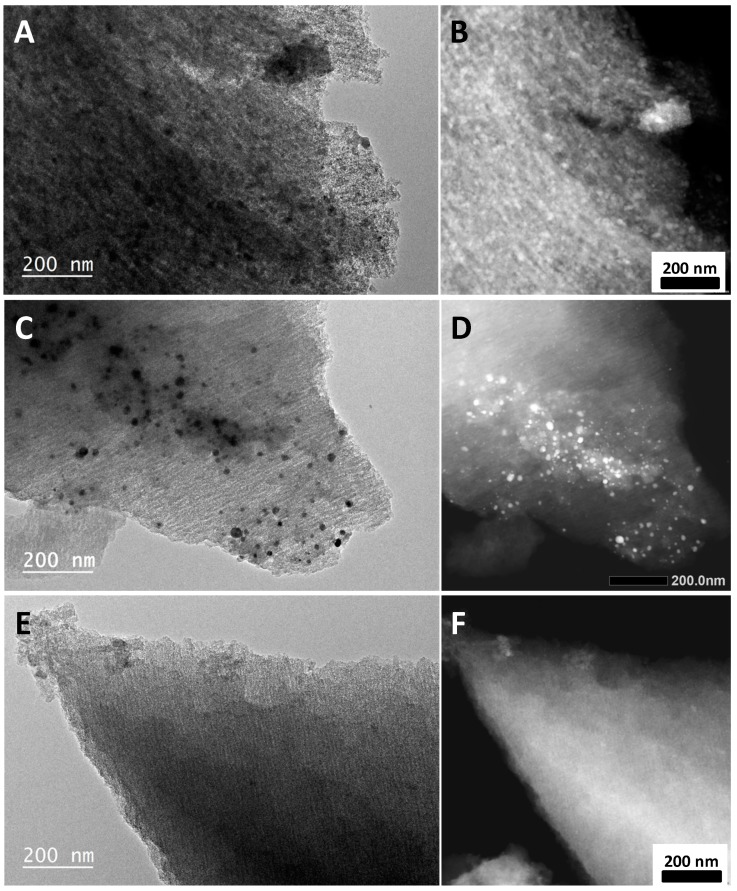
(**left**) Classical TEM and (**right**) corresponding STEM pictures of C/NiHCFe (**A**,**B**), C/CuHCFe (**C**,**D**) and C/InHCFe (**E**,**F**).

The influence of metal type on the formation of C/MHCFe nanocomposites and their adsorption capacities was examined first. General morphological and structural characteristics of the nanocomposites were obtained by TEM and STEM studies (Figure 1). Nanoparticles were not observed on classical TEM pictures on C/NiHCFe and C/InHCFe materials. As the contrast in STEM images depends on atomic number Z, the metal phases will appear brighter than carbon matrix. This behavior facilitates localization of metal nanoparticles in a carbon matrix. The STEM mode showed that the metal was present in both nanocomposites C/NiHCFe and C/InHCFe in a very diffuse manner. This might be related to a high density of particles in the carbon matrix. The morphology of C/CuHCFe was quite different with nanoparticles clearly observable in both classical TEM and STEM techniques. They were dispersed rather heterogeneously in the carbon network and their size varied between 10 and 30 nm.

**Figure 2:**
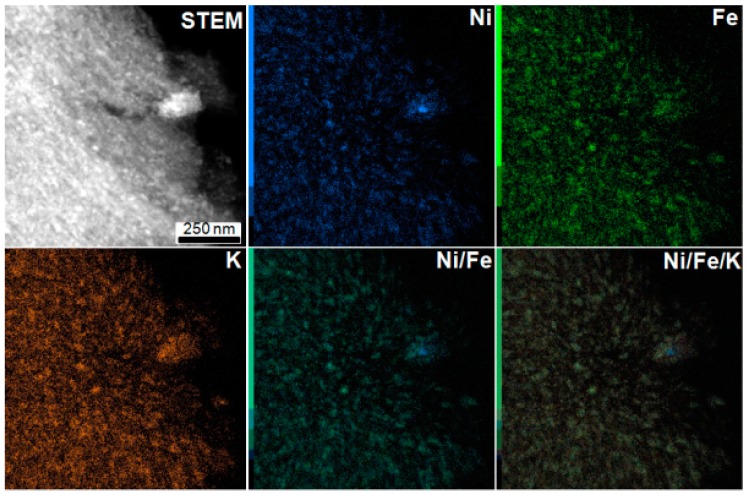
EDX mapping of the C/NiHCFe material showing the nickel, iron, and potassium presence in the nanoparticles. Superposition of Ni and Fe and Ni, Fe, and K mapping.

EDX mapping was performed to get more information about the local chemical composition of the particles. As an example, Figure 2 presents the EDX mapping of C/NiHCFe material, while those of C/CuHCFe and C/InHCFe are provided in Figures S1 and S2 (Supporting Information, SI). As observed in Figure 2, the presence of nickel and iron, but also potassium, was detected in the material. The superposition of the EDX mapping of these three metals revealed perfect matching, indicating the presence of all these metals in the nanoparticle structure. A large particle placed in the extremity of the carbon is observed as well in addition to the small particles and seems to be richer in Ni than the other metals. For C/CuHCFe and C/InHCFe (Figures S1 and S2) the presence of potassium was also revealed in the particle structure.

**Figure 3:**
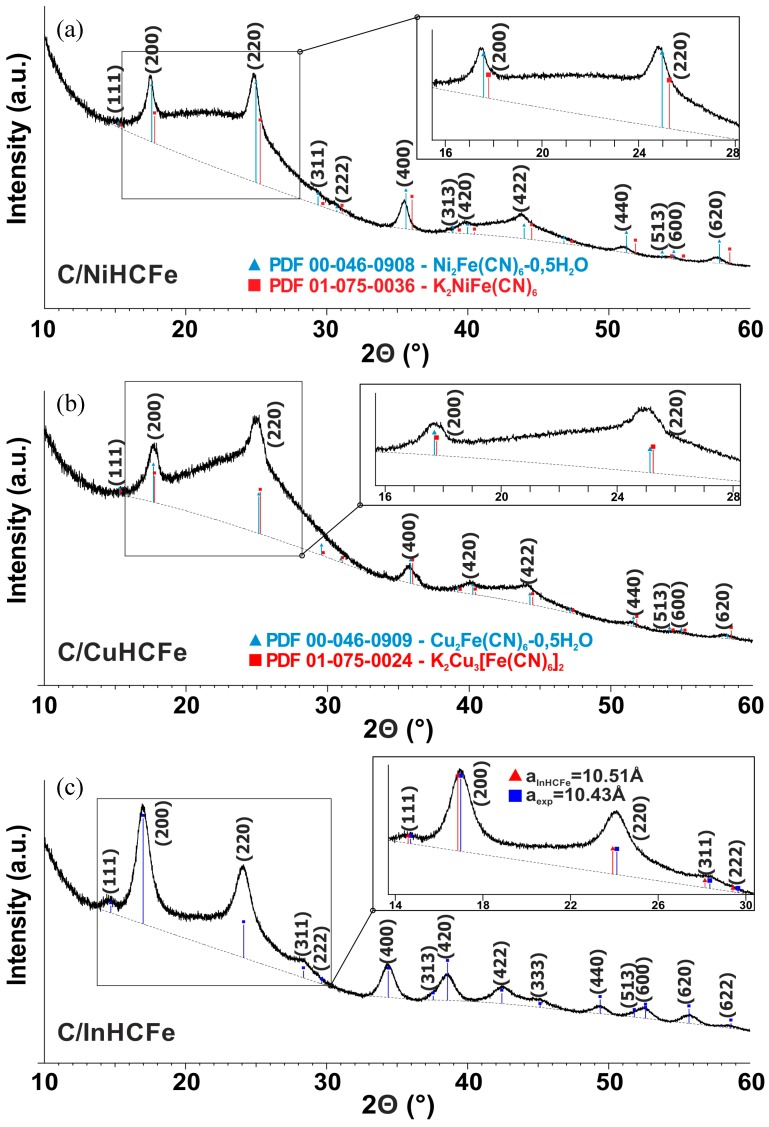
X-ray diffractrograms (CuKα_1,2_) for C/NiHCFe (**a**), C/CuHCFe (**b**), and C/InHCFe (**c**). Background (default parameters for curvature and threshold in DIFFRAC.EVA) is the dotted line for each composite. Corresponding possible PDF patterns are indicated, the inserts allowed for a better view of details in the different pattern lines position with the most intense peaks (200) and (220). For the InHCFe phase, theoretical peaks positions were calculated from Reference [34] and then slightly shifted (unit cell parameter a near ≈10.43 Å).

The structure of the materials was determined by XRD technique and the recorded diffractograms are presented in Figure 3. According to the rather large FWHM (full width at half maximum) observed for each synthesized material, a search-match process from XRD data via DIFFRAC.EVA [33] led to several possible phases when corresponding patterns were available in the International Centre for Diffraction Data (ICDD) database.

For C/NiHCFe (Figure 3a), the XRD data matched with the powder diffraction file (PDF )to lead to two possible patterns for identification: PDF N°00-046-0908 (Ni_2_Fe(CN)_6_-0.5H_2_O, F–43m, a = 10.077 Å) and PDF N° 01-075-0036 (K_2_NiFe(CN)_6_, F–43 m, a = 9.96 Å). The observed FWHMs were narrower than in the Cu compound, and the reflection positions better split because of the unit cell parameters were slightly different. Consequently, a better matching was observed for the potassium-free NiHCFe. However, the discrimination of one phase versus the other only on the basis of XRD remained difficult because the experimental FWHM was too large to identify unambiguously. However, taking into account the EDX results showing the presence of potassium in the materials, the potassium containing NiHCFe (K_2_NiFe(CN)_6_) phase could not be excluded. 

The same observation is made for C/CuHCFe (Figure 3b). XRD data match with PDF N°00-046-0909 (Cu_2_Fe(CN)_6_-0.5H_2_O, F–43m, a = 10.013 Å), and also with PDF N°01-075-0024 (K_2_Cu_3_[Fe(CN)_6_]_2_, F–43m, a = 9.97 Å). 

This was clearly illustrated with both most intense peaks (200) and (220) (Figure 3); according to these PDFs, the (200) and (220) reflection positions for potassium and potassium-free CuHCFe only differed by 0.080° 2θ and 0.111° 2θ, respectively. This was between eight up to nearly ten times smaller than the corresponding observed FWHM for these two peaks: 0.64° 2θ and 1.06° 2θ. Therefore, XRD data were not efficient enough to discriminate between these two phases. Complementary EDX mapping analysis on particles provided in the SI (Figure S1) confirmed the presence of potassium in the C/CuHCFe material, in agreement with the XRD data showing the presence of both potassium and potassium free CuHCFe. 

In the case of C/InHCFe (Figure 3c), due to the absence of indium hexacyanoferrate pattern in ICDD, Crystallography Open Date Base (COD), or International Center for Diffraction Data (ICSD) databases [35], the phase identification required a specific search/match strategy. The crystal structure of InHCFe was recently reported by Chen et al. [34], which allowed for the import of the corresponding CIF (crystallographic information file) into DIFFRAC.EVA and calculate the associated pattern (In_2_Fe(CN)_6_, Fm–3m, a = 10.51 Å). This pattern confirmed the presence of the InHCFe phase in the nanocomposite (Figure 3), but one notices a slight shift to highest angles of the experimental phase versus the pattern reflection positions. This could be an indication of the presence of potassium inside this InHCFe compound because, as observed with the corresponding Ni or CuHCFe phases, the presence of K inside the crystal structure decreased the unit cell parameter (from 10.013 Å to 9.97 Å (≈ 0.4%) for Cu and from 10.077 Å to 9.96 Å (≈1.1 %) for Ni). Our experimental unit cell parameter for In was around 10.43 Å, which was ≈0.7 % from the K-free InHCFe (10.51 Å). Therefore, this pattern shift could have been related to the presence of potassium and further confirmed by the EDX mapping results. Individual peak profile fitting results via WinPlotr [36], after Bruker raw data conversion with PowDLL [37], were used with the Scherrer formula [38] that can provide some qualitative estimation of the (coherently scattering) domain size to be employed for comparison purpose in the case of our three nanocomposites. Using (200) and (220) peaks (stronger intensity), we obtained an average apparent (coherently scattering) crystallite size of 12.4 nm for C/NiHCFe, 8.3 nm for C/CuHCFe, and 6.3 nm for C/InHCFe.

**Figure 4:**
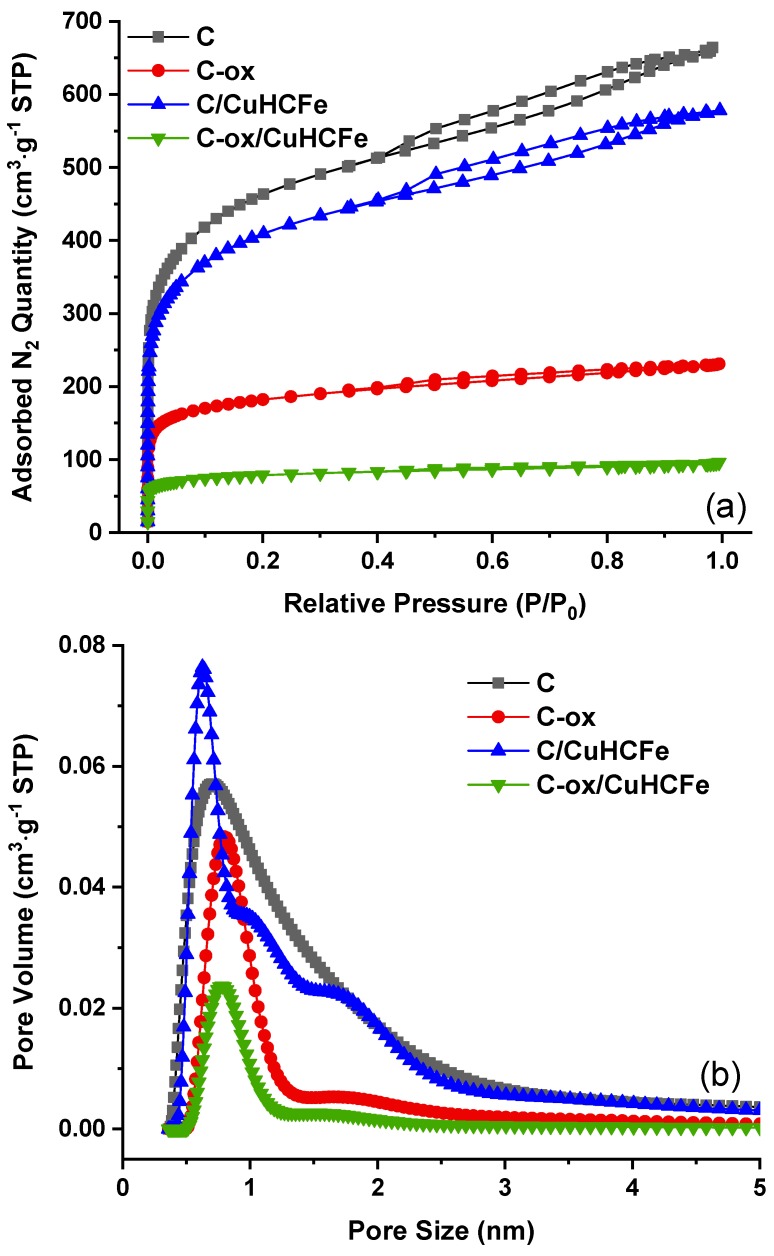
Nitrogen adsorption isotherms (**a**) and pore size distribution (**b**) of C, C-ox, C/CuHCFe, and C-ox/CuHCFe. The pore size distribution was calculated using the 2-D non-local density functional theory.

The textural features of the materials were determined using nitrogen adsorption/desorption measurements and the corresponding isotherms were presented in Figure 4, while textural data extracted from isotherms were presented in Table 1. 

The isotherms were of type I specific to microporous materials (pore size < 2 nm), but a small hysteresis was observed indicating small mesopores. The specific surface area of LW27 carbon was 1643 m^2^·g^−1^, while the total pore volume was 0.87 cm^3^·g^−1^, distributed between micropores (0.49 cm^3^·g^−1^) and mesopores (0.38 cm^3^·g^−1^). The textural values (Table 1) showed lower porous characteristics (specific surface area and pore volumes) for the nanocomposites compared to the activated carbon L27W due to the presence of the metallic phase in the porous structure.

The decrease of these characteristics is more pronounced for C/InHCFe and C/CuHCFe, and both microporosity and mesoporosity are affected. We tried to correlate this decrease of the textural values to the amount of particles in the composite. TGA under air was used (Figure S3, Supporting Information) which allow to oxidize (burn) the carbon and to oxidize the metallic particle and further determine the amount of metal oxide residue formed. In the case of C/InHFe, the amount of oxides was higher, i.e., 19.5 wt.% (Table 1) compared to C/NiHFe (8.3 wt.%) and C/CuHFe (9.2 wt.%), which exhibited similar values. It is worth noting that due to the different nature of the metals the amount of oxides determined by TGA cannot be directly linked to the initial amount of the metals in the material. This partly explains why we could observe a direct correlation between the oxide amounts and the decrease of the textural values. However, this decrease of the porous characteristics was not very important, indicating a good accessibility from the metal phase to the surface. The increased density of these materials after impregnation might have also influenced the calculation of these characteristics. We have observed a more important specific surface area in the case of C/NiHCFe compared to the activated carbon. This behavior might be linked to the presence of nanoparticles outside the porosity, thus increasing the specific surface area due to their important surface/volume ratio.

**Figure 5:**
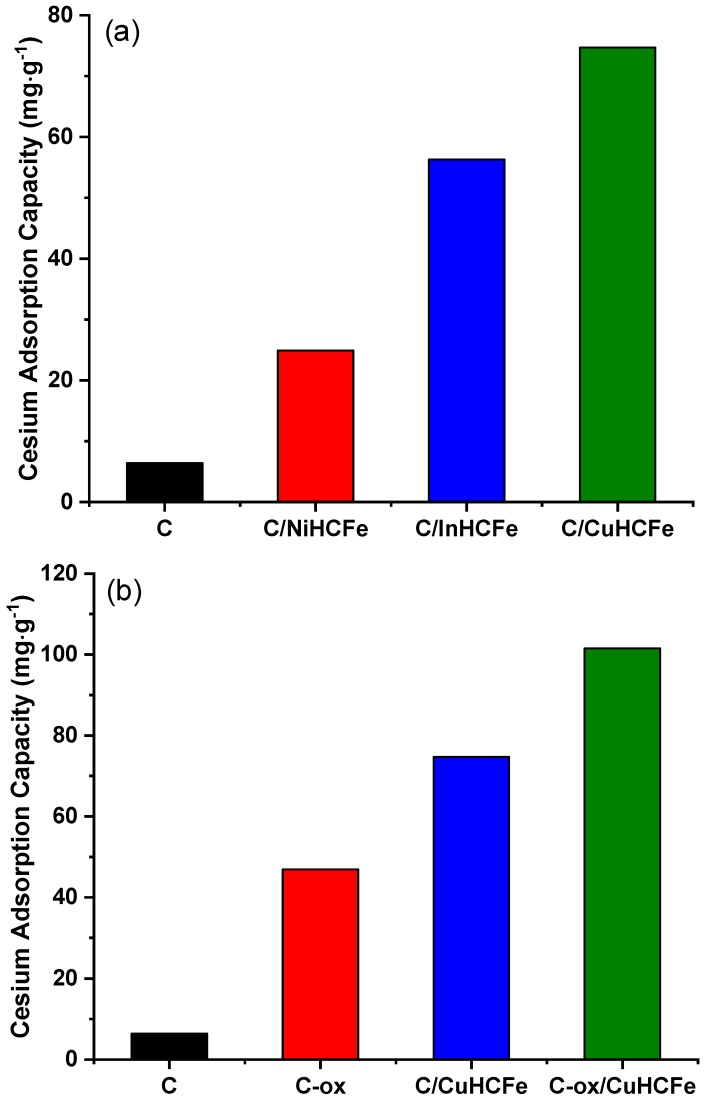
Cesium adsorption capacities measured with atomic absorption spectroscopy on (**a**) three C/MHCFe nanocomposites and the C reference, and (**b**) both pristine and oxidized carbon supports and their corresponding CuHCFe nanocomposites.

The removal of cesium from aqueous solution with these materials was done using a batch adsorption process. These measurements were performed at room temperature and the contact time was fixed at 24 hours. The samples’ concentration used in the contact solution was fixed at 1 g·L^−1^. Thereby, we established a contact during 24 hours between 50 mg of nanocomposite and 50 mL of cesium stock solution. The cesium adsorption capacities, measured using atomic absorption, are presented in Figure 5. They show a low cesium adsorption capacity for the activated carbon C (6.4 mg·g^−1^). For the C/NiHCFe composite, the cesium adsorption activity was improved four-fold (24.9 mg·g^−1^) compared to pristine carbon, but still remained low compared to other adsorbents [2,39]. The adsorption capacities obtained on C/InHCFe and C/CuHCFe were further increased to 56.3 mg·g^−1^ and 74.7 mg·g^−1^, respectively. The comparison between L27W carbon and the C/metal-based nanocomposites adsorption capacities led to the conclusion that carbon textural properties, such as specific surface area and pore volume, were not governing factors in the cesium adsorption process. However, the metallic phase present in the nanocomposites clearly had a positive influence on the adsorption capacity.

It is now interesting to find a way to increase these performances. Therefore, we chose to modify the surface chemistry of the L27W activated carbon as a way to increase the dispersion and the charge of metal hexacyanoferrate adsorbed on the carbon. 

### 3.2. Influence of the Carbon Surface Chemistry

The surface chemistry of the support has an important role in a nanocomposite synthesis through impregnation process. The nature and the concentration of oxygen surface functional groups may impact the nanoparticles formation as well as the Cs adsorption. The surface functional groups can be modified using thermal and chemical treatments. Oxidation in gas or liquid phase can be applied to increase the concentration of oxygen groups present at the surface. Figueiredo et al. [40] used a nitric acid treatment to increase the concentration of carboxyl groups (–COOH). Activated carbon (L27W)/copper hexacyanoferrate nanocomposite was selected to examine the effect of surface chemistry, taking into consideration its highest performance in cesium adsorption among the synthesized nanocomposites. 

The atomic composition quantification of both the pristine L27W carbon matrix and nitric acid modified carbon were measured using two techniques: X-ray photoelectron spectroscopy (XPS) and energy dispersive X-ray spectroscopy (EDX). The XPS technique allowed for the determination of the atomic quantification in the surface of the material (≈10 nm), while the EDX analysis provided the bulk composition of the material. 

**Figure 6:**
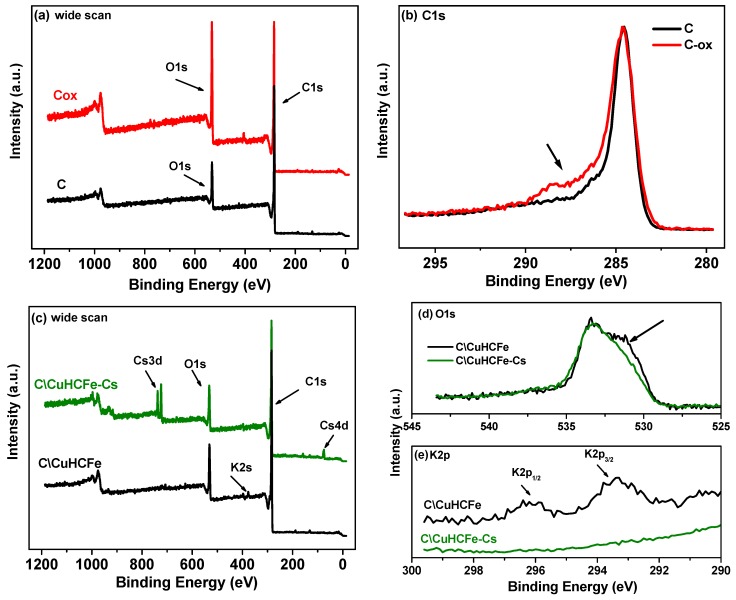
Wide scan (**a**) and C1s (**b**) core level XPS spectra of C and C-ox supports. Wide scan (**c**), O1s (**d**), and K2p (**e**) core level XPS spectra of C/CuHCFe and C/CuHCFe-Cs.

The wide XPS spectra (Figure 6a) show the increase of the intensity of the oxygen O1s peak for C-ox compared to C. The increase in the oxygen content for C-ox can be seen in the high-resolution spectra of the C1s peak (Figure 6b) due to the increase of the chemical carbon–oxygen bonds situated between 285.5 eV and 290 eV, as indicated by an arrow. The deconvolution of the high-resolution C1s spectra revealed the presence of several types of oxygen functional groups for the C material, i.e., hydroxyl/ether (C-OR, 4.4 at.%), carbonyl (C=O, 2.2 at.%), and carboxyl (O=C-O, 1.9 at.%). The acidic treatment significantly increased the amount of all these groups for C-ox material to 6.7 at.%, 5.2 at.%, and 5.1 at.%, respectively.

The atomic composition is presented in Table 2, and it can be seen that C-ox presented a higher amount of oxygen, 23.4 at.% versus 12.9 at.% for C, due to the nitric acid treatment. Nitrogen was detected in addition on C-ox due to the nitric acid treatment. The detected phosphor was a result of the chemical activation process of L27W with phosphoric acid. The nitric acid treatment tends to decrease this concentration in C-ox from 1.2% to 0.2 at.%. The composition results were found rather similar with those obtained using the EDX technique (see Table S1, Supporting Information) indicating that the composition was the same in the surface and in the core of the materials. 

The X-ray diffractograms (Figure S4) of both C/CuHCFe and C-ox/CuHCFe nanocomposites proved the formation of similar copper hexacyanoferrate phases were as previously described. Therefore, the oxidized carbon support did not induce modification of the metallic phase formation. A higher concentration in copper hexacyanoferrate in the C-ox/CuHCFe sample was found in the TGA results (see Table 1), with the final weight of copper-based residues being 13.6% for C-ox/CuHCFe versus 9.2% for C/CuHCFe. 

It was also important for the influence of the nitric treatment on the material porosity to be evaluated. The results of nitrogen adsorption on both supports (C and C-ox) and their corresponding nanocomposites are presented in Table 1. A drastic decrease of porous characteristics of the carbon matrix after the nitric acid treatment was observed. The surface area decreased from 1643 m^2^·g^−1^ to 573 m^2^ ·g^−1^, while the total pore volume decreased from 0.87 cm^3^·g^−1^ to 0.31 cm^3^·g^−1^. The microporosity and mesoporosity both decreased as well. In addition, the textural characteristics were further decreased following the impregnation process. 

Nitrogen adsorption isotherms and pore size distribution of these samples are presented in Figure 4. While isotherms are of type I/IV for C and C/CuHCFe samples combining microporosity and mesoporosity, the mesoporous part of these curves was difficult to observe on C-ox and almost disappeared for the C-ox/CuHCFe sample, illustrating the decrease of mesoporosity. This observation is in line with the pore size distribution curves, further showing the decrease of the pore volume and size. In addition, we noticed that the incorporation of the CuHCFe phase led in both cases to a narrow pore size distribution. This might indicate the presence of some particles in the carbon pores. It is now very interesting to see whether the decrease of porous characteristics and the increase in the concentration of oxygen functional groups affect the cesium adsorption performances.

Cesium adsorption measurements were done using atomic absorption spectroscopy following the same process described above. The cesium adsorption capacity of the L27W carbon obtained after the nitric acid treatment showed C-ox to be significantly higher (46.9 mg·g^−1^, Figure 5b) than the cesium adsorption capacity of L27W carbon, C (10.2 mg·g^−1^, Figure 5b). The adsorption capacity measured for oxidized carbon was very high compared to many other works summarized in Khandarek et al. [41]. In that paper, the highest reported adsorption capacity was 55.5 mg·g^−1^ for 1000 mg·L^−1^ solution of Cs in water. If one compares the adsorption capacity for similar concentration of Cs as in our work, i.e., 265 mg·L^−1^, reported values in the literature are ≈25 mg·g^−1^, which are two times lower than our values. The better performances of our materials can be explained by the higher amount of oxygen introduced via chemical wet modification (≈ 13 at.%) compared to 9 at.% in the work of Khandrarek et al. [41] where air oxidation at 300 °C was performed. When our material was modified by air oxidation at 350 °C, it indeed showed lower adsorption capacity values as well (17.7 mg·g^−1^) compared to the one modified by chemical oxidation (46.9 mg·g^−1^).

This result shows that the surface chemistry of an activated carbon is a parameter strongly influencing the cesium adsorption capacity, more than its porous characteristics (specific surface area, pore volume), which were drastically reduced by this treatment (see Table 1). Therefore, physisorption on Cs in the carbon porosity via weak bonds was not enough to achieve high adsorption capacity. It is more likely that Cs interacted via chemical bonds with the observed oxygen functional groups using XPS (C–OR (R = C or H), C=O, O–C=O). This was sustained by the decrease of the oxygen content from 23.4 at.% for C-ox to 21.5 at.% after Cs adsorption (C-ox-Cs, Table 2). A slight decrease in the nitrogen content (–NO^-^_3_ groups) from 2.3 to 1.9 at.% was noticed as well.

The nanocomposite obtained using oxidized carbon (C-ox/CuHCFe) as a support also possessed a better cesium adsorption capacity (101.5 mg·g^−1^, Figure 5b) than the nanocomposite (C/CuHCFe) obtained from unmodified carbon (74.7 mg·g^−1^, Figure 5b). However, this important adsorption capacity was slightly contrasted with the already important Cs adsorption capacity observed on the oxidized carbon (46.9 mg·g^−1^), whereas the copper residues weight measured in TGA were more important in C-ox/CuHCFe than in C/CuHCFe, indicating a higher metal concentration in C-ox/CuHCFe. Therefore, we could expect an increase of cesium adsorption capacities to be more important in the case of C-ox/CuHCFe. This might be related to CuHCFe nanoparticles, which via functional groups, are fixed to the carbon structure. Such functional groups are no longer available that would have otherwise contributed to cesium adsorption capacities. Such a hypothesis is sustained by the XPS results, showing a decrease in the oxygen content from 23.4 at.% for C-ox to 17.8 at.% for C-ox/CuHCFe; therefore, some oxygenated groups were removed from the carbon surface to leave a place for metal salts to anchor, most probably in the carbon defects (Table 2). 

Although it is difficult to compare these results in a rigorous manner with those reported in other publications due the differences on the methodologies of cesium adsorption testing, an attempt is done as presented in Table 3. The adsorption capacity corresponding to different materials extracted from the literature allow one to first notice that a cesium adsorption capacity above 100 mg·g^−1^, which is very interesting. Values higher than 150 mg·g^−1^ are reported only for composites containing a polymer phase (P4VB, PVA, PRG) in addition to carbon and metal hexaceyanoferrate phase; however, such polymers do not present high chemical and mechanical stability as is advantageously provided by carbon.

To get more insights in the adsorption mechanism of Cs, XPS analyses were performed on C/CuHCFe composite materials before and after Cs adsorption (Figure 6c–e and Table 2). 

The wide XPS spectra (Figure 6c) of C/CuHCFe and C/CuHCFe-Cs show that after the Cs adsorption process there was a decrease of the oxygen peak, the disappearance of potassium peak, and the appearance of the Cs peak. The O1s spectra of C/CuHCFe and C/CuHCFe-Cs (Figure 6d) reveals the decrease of the chemical carbon–oxygen bonds, i.e., C–O, C=O and O=C–O (situated between 530 eV and 533 eV and indicated by an arrow). Moreover, the Figure 6e spectra show (in the case of C/CuHCFe) the presence of the K2p peak having two components K2p_3/2_ and K2p_1/2_ with a spin-orbit coupling of 2.8 eV. This K2p peak disappeared in the case of the C/CuHCFe-Cs material.

The potassium amount present in (C/CuHCFe) was 1.1 at.% (Table 2), while after Cs adsorption (C/CuHCFe-Cs), no potassium was detected, and only Cs was observed (0.93 at.%). These results are consistent with an ion-exchange mechanism of K^+^ and Cs^+^ reported in previous studies [18,46,47], which could explain why potassium was not detected after the cesium adsorption. The slight decrease of the copper concentration might also be associated to a partial replacement of copper by cesium at the sample surface. By using copper ferrocyanide and copper ferricyanide phases (exempt of monovalent cation), Ayrault et al. [13] proposed a complex Cs adsorption mechanism involving in a first step the diffusion of ion pairs (Cs^+^, NO_3_^-^) followed by the formation of a new crystalline phase and copper release. Han et al. [48] used copper ferrocyanide and proposed an ion exchange mechanism of Cu^2+^ with Cs^+^. In the case of our material, the ion exchange of Cu with Cs might be possible due to the presence of a pure copper phase. According to Vincent et al. [18], the synthesis of metal cyanoferrate usually leads to a mixture of materials with different structures and compositions exhibiting more complex adsorption mechanisms.

Therefore, the XPS results revealed that the Cs adsorption capacity of C-ox was governed by the carbon oxygen functional groups/defects while in the case of C/CuHCFe was more related to a “cation exchange” mechanism. The adsorption capacities of these materials were found to be 46.9 mg·g^−1^ for C-ox and 74.7 mg·g^−1^ for C/CuHCFe. By combining the C-ox with CuHCFe, the resulting nanocomposites (C-ox/CuHCFe) presented a Cs adsorption capacity that was higher (101.5 mg·g^−1^) than the ones delivered by C/CuHCFe and C-ox, but still a bit lower than the one we could expect through an eventual cumulative effect between these two materials. As pointed out by XPS, this effect can be understood via the utilization of some functional groups to attach the CuHCFe nanoparticles, which therefore are not available anymore for cesium adsorption. 

Nevertheless, such an association between an oxidized activated carbon support and a metal hexacyanoferrate phase show promise for the development of new efficient absorbents.

## 4. Conclusions

Novel nanocomposite materials combining a commercial activated carbon and a transition metal (Ni, In, Cu) hexacyanoferrate phase using a very simple impregnation process have been synthesized in this work. The nanocomposites present similar porosity, but different metal hexacyanoferrate nanoparticle size and distributions. Among these materials, the nanocomposite C/CuHCFe exhibited the best cesium adsorption performances (74.7 mg·g^−1^). Hence, the surface chemistry of the activated carbon was modified with a nitric acid treatment in order to make it more suitable as a support for these metal hexacyanoferrate phase. Cs adsorption tests showed that adsorption capacities were among the highest reported yet for a carbon material (46.9 mg·g^−1^). This behavior could be related to the oxygen-rich surface chemistry of the carbon, which was a far more crucial parameter favoring cesium adsorption than the porous characteristics. In addition, the cesium adsorption capacity obtained with C-ox/CuHCFe nanocomposite was greatly improved compared to their counterpart’s nanocomposites using unmodified carbon support (≈100 mg·g^−1^). Such an improvement of the Cs adsorption capacity was associated with a double-type absorption mechanism involving Cs “cation exchange” with the metal hexacyanoferrate phase and Cs adsorption on the carbon–oxygen functional groups. These performances might be further increased by tuning the surface chemistry and metal phase loading of these materials and a cheap commercial nanocomposite for cesium-contaminated water could become feasible.

## Figures and Tables

**Table 1:** Textural characteristics of carbon supports and C/MHCFe nanocomposites extracted from nitrogen adsorption isotherms; the amount of metal-oxide residue obtained using TGA analyses and Cs adsorption capacities.

Material	S_BET_ (m^2^·g^−1^)	V_total pores_ (cm^3^·g^−1^)	V_micropores_ (cm^3^·g^−1^)	V_mesopores_ (cm^3^·g^−1^)	TGA Residue (wt%)	Cs Capacity (mg·g^−^^1^)
C	1643	0.87	0.49	0.38	6.9	6.4
C/NiHCFe	1799	0.85	0.55	0.30	8.3	24.9
C/InHCFe	1342	0.65	0.41	0.24	19.5	56.3
C/CuHCFe	1450	0.74	0.43	0.31	9.2	74.7
C-ox	573	0.31	0.21	0.10	4.2	46.9
C-ox/CuHCFe	246	0.12	0.09	0.03	13.6	101.5

Key to abbreviations: C—activated carbon; C-ox—oxidized carbon with nitric acid; NiHCFe—nickel hexacyanoferrate; InHCFe—indium hexacyanoferrate; CuHCFe—copper hexacyanoferrate.

**Table 2:** XPS atomic quantification (at.%) of carbon supports and carbon/CuHCFe materials before and after the Cs adsorption.

Materials	C1s	O1s	K2p	N1s	P2p	Cu2p3/2	Fe2p3/2	Cs3d
C	85.9	12.9	-	-	1.2	-	-	-
C-ox	73.8	23.4	-	2.3	0.2	-	-	-
C/CuHCFe	82.9	13.4	1.2	1.1	1.0	0.24	0.15	-
C-ox/CuHCFe	68.5	17.8	3.2	7.6	-	1.7	1.05	-
C/CuHCFe-Cs	84.5	12.6	-	1.0	0.7	0.12	0.15	0.91
C-ox-Cs	75.4	21.5	-	1.9	-	-	-	1.16

**Table 3:** Cs^+^ adsorption capacity extracted from the literature for different materials.

Adsorbent Type	Adsorbent Name	Sorption Capacity (mg·g^−1^)	Reference
	Activated Carbon	6	[28]
Carbon	Oxidized MWCNTs	12.75	[42]
	Oxidized bamboo charcoal	53.5	[41]
	C-ox	46.9	This study
	NiHCFe-AC(R)	31.2	[30]
	AC/KHCFe	1.38	[27]
Carbon/MHCFe	AC/CuHCFe	61.2	[28]
	AC/KNiCFe	163.9	[29]
	C-ox/CuHCFe	101	This study
	MWCNTs-P4VB-CuHCFe	150	[43]
Carbon/Polymer/HCFe	SWCNTs-PRG-CuHCFe	240	[44]
	Graphene Oxide-PVA-CuHCFe	164.5	[45]

Key to abbreviations: MHCFe—metal hexacyanoferrate; NiHCFe—nickel hexacyanoferrate; KHCFe—potassium iron hexacyanoferrate; CuHCFe—copper hexacyanoferrate; KNiCFe—potassium nickel hexacyanoferrate; AC—activated carbon; MWCNTs—multi wall carbon nanotubes; SWCNTs—single wall carbon nanotubes; P4VB—poly(4-vinylpyridine); PRG—propargylamine; PVA—poly vinyl alcohol.

## Supplementary Materials

The following are available online at https://www.mdpi.com/1996-1944/12/8/1253/s1, Figure S1: EDX mapping of C/CuHCFe material showing the copper, iron and potassium presence in the nanoparticles; Figure S2: EDX mapping of C/InHCFe material showing the potassium, indium and iron presence in the nanoparticles; Figure S3: TGA analyses under air on C/HCFe nanocomposites (a) the weight loss and (b) derivative weight loss; Figure S4: X-ray diffractograms of C/CuHCFe and Cox/CuHCFe. Vertical bars indicate peaks position of the related phase Cu_2_Fe(CN)_6_·0.5H_2_O and K_2_Cu_3_[Fe(CN)_6_]_2_; Table S1: Atomic quantification of pristine C and C-HNO_3_ modified carbon matrix.
