# Supplementary material for: Activated Carbon/Transition Metal (Ni, In, Cu) Hexacyanoferrate Nanocomposites for Cesium Adsorption
Source: Materials (Basel). 2019 Apr 16;12(8):1253. doi: 10.3390/ma12081253 (PMC6514891; doi:10.3390/ma12081253)
Supplement: Supplementary file 1 [file materials-12-01253-s001.pdf]

materials

MDPI

Article

Activated Carbon / Transition Metal (Ni, In, Cu) Hexacyanoferrate Nanocomposites for Cesium Adsorption

Julien Kiener [1,2], Lionel Limousy [1,2], Mejdi Jeguirim [1,2], Jean-Marc Le Meins[1,2], Samar Hajjar-Garreau [1,2], Gaetan Bigoin [3], and Camélia Matei Ghimbeu [1,2,*]

[1] Université de Haute-Alsace, CNRS, Institut de Science des Matériaux de Mulhouse (IS2M) UMR 7361, F-68100 Mulhouse, France; julien.kiener@yahoo.com (J.K.); Lionel.limousy@uha.fr (L.L.); Mejdi.jeguirim@uha.fr (M.J.); Jean-Marc.Le-meins@uha.fr (J.M.); samar.hajjar@uha.fr (S.H.)

[2] Université de Strasbourg, F-67081 Strasbourg, France

[3] ONET Technologies, 36 Bd de l'Océan-CS 20280, 13258 Marseille, France; gbigoin@onet.fr

* Correspondence: camelia.ghimbeu@uha.fr; Tel.: +33 (0) 3 89 60 87 43

Received: 22 March 2019; Accepted: 12 April 2019; Published: date

Table 1. Atomic quantification of pristine C and C-HNO_3 modified carbon matrix.

Element / Material	XPS quantification (at. %)		EDX quantification (at. %)	
	**C**	**Cox**	**C**	**Cox**
C	85.9	73.8	84.8	75.9
O	12.9	23.4	14.4	23.6
P	1.2	0.2	0.8	0.6
N	-	2.3	-	-

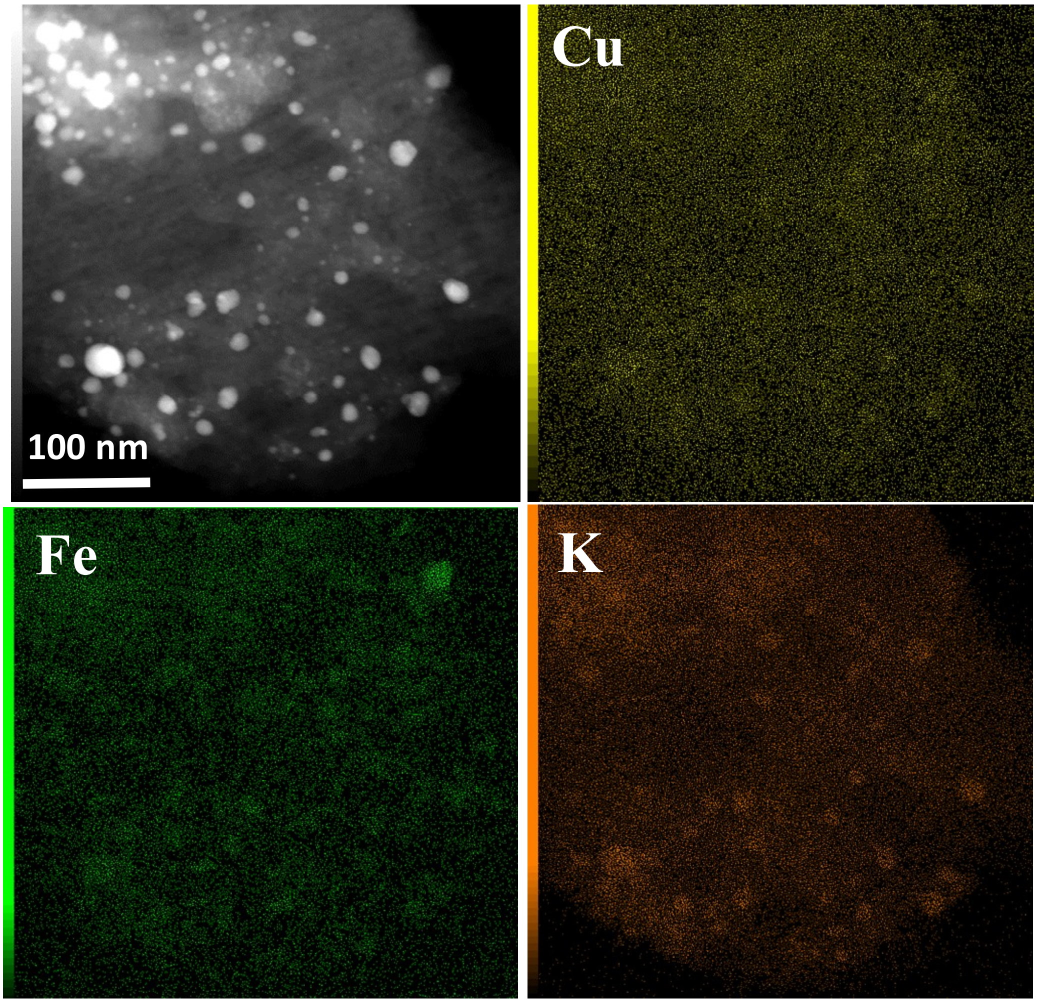

Figure 1. EDX mapping of C/CuHCFe material showing the copper, iron and potassium presence in the nanoparticles.

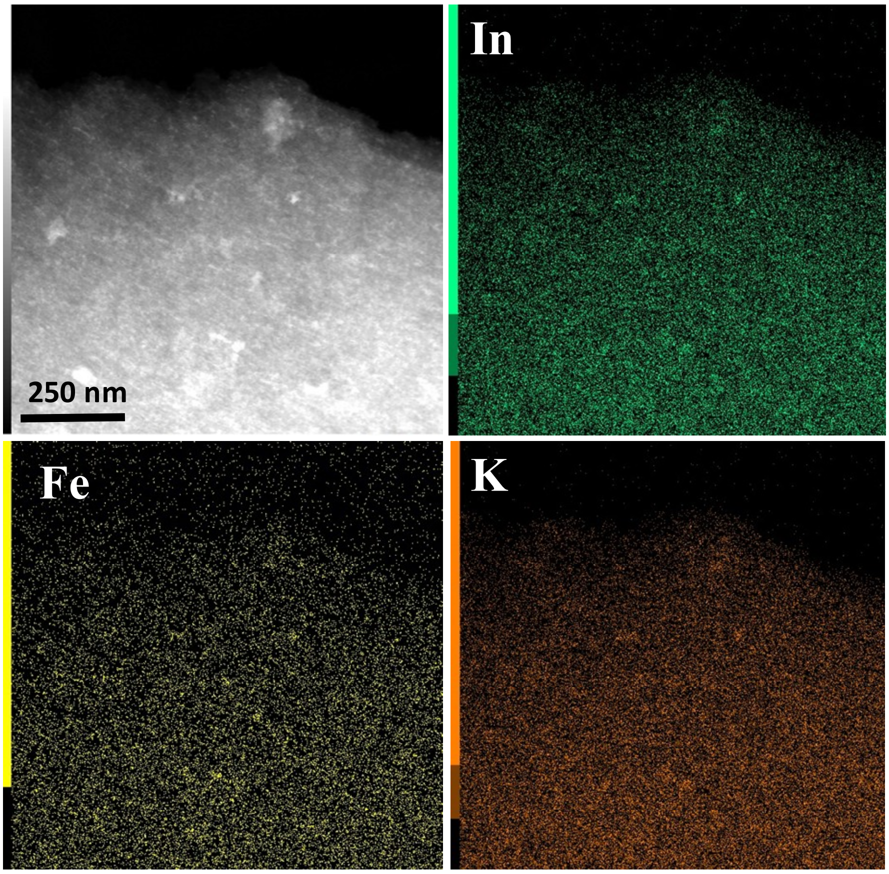

Figure 2. EDX mapping of C/InHCFe material showing the potassium, indium and iron presence in the nanoparticles.

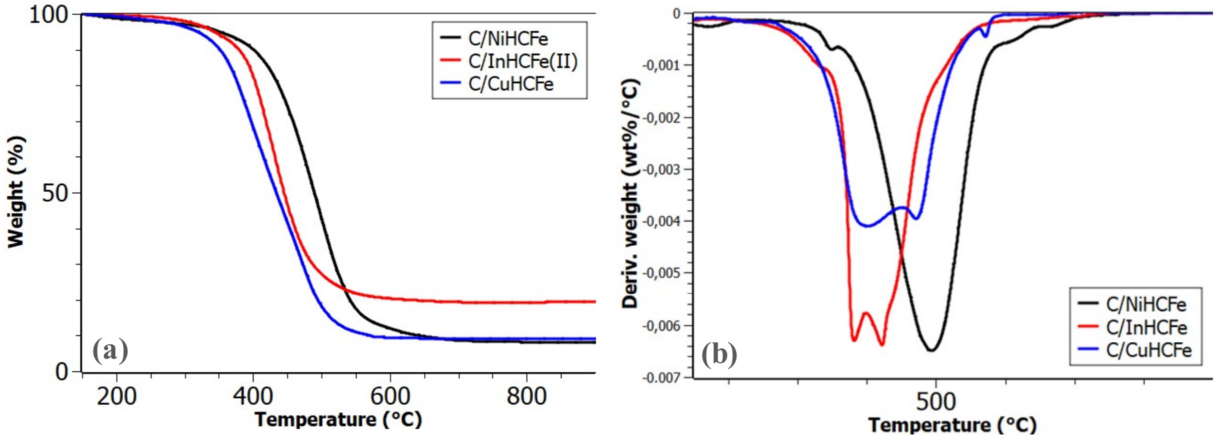

Figure 3. TGA analyses under air on C/HCFe nanocomposites (a) the weight loss and (b) derivative weight loss.

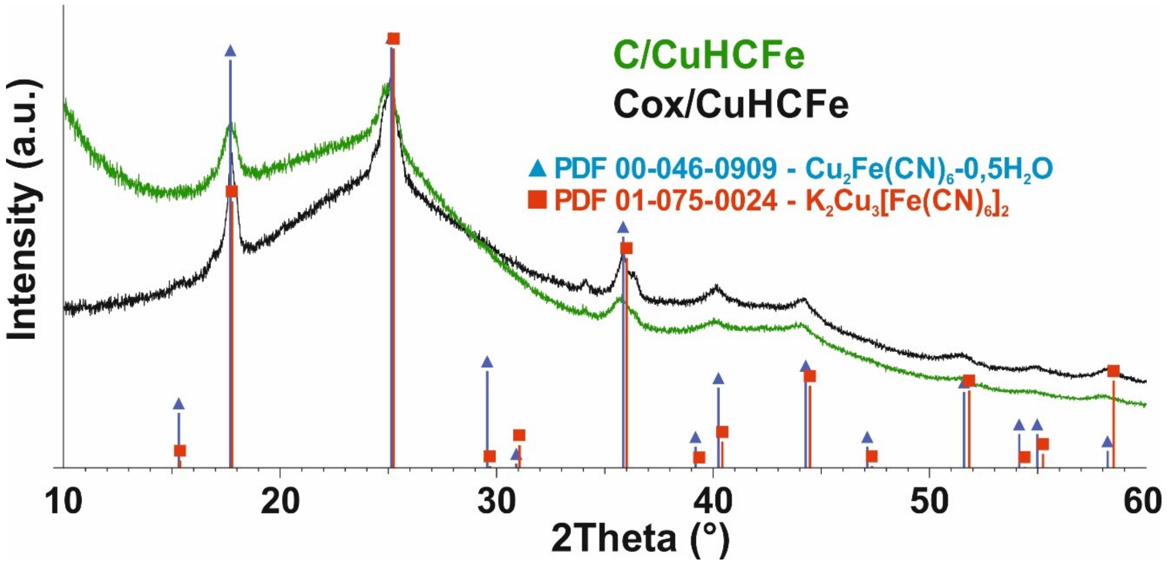

Figure 4. X-ray diffractograms of C/CuHCFe and Cox/CuHCFe. Vertical bars indicate peaks position of the related phase $Cu_2Fe(CN)_6 \cdot 0.5H_2O$ and $K_2Cu_3[Fe(CN)_6]_2$.
